# Novel Antidepressant-Like Activity of Caffeic Acid Phenethyl Ester Is Mediated by Enhanced Glucocorticoid Receptor Function in the Hippocampus

**DOI:** 10.1155/2014/646039

**Published:** 2014-11-16

**Authors:** Mi-Sook Lee, Young Han Kim, Bo-ram Lee, Seung-Hae Kwon, Won-Jin Moon, Kwan-Su Hong, Yun Seon Song, Kyoji Morita, Dae Hyun Hahm, Insop Shim, Song Her

**Affiliations:** ^1^Division of Bio-Imaging, Chuncheon Center, Korea Basic Science Institute, Chuncheon 200-701, Republic of Korea; ^2^Department of Science in Korean Medicine, Graduate School, College of Korean Medicine, Kyung Hee University, Seoul 130-701, Republic of Korea; ^3^Gwangju Center, Korea Basic Science Institute, Gwangju 500-757, Republic of Korea; ^4^MR Research Center, Korea Basic Science Institute, Cheongwon 363-883, Republic of Korea; ^5^College of Pharmacy, Sookmyung Women's University, Seoul 140-742, Republic of Korea; ^6^Laboratory of Neuropharmacology, Department of Nursing, School of Health Sciences, Shikoku University, Tokushima 771-1192, Republic of Korea; ^7^Acupuncture & Meridian Science Research Center, College of Oriental Medicine, Kyung Hee University, Seoul 130-701, Japan

## Abstract

Caffeic acid phenethyl ester (CAPE) is an active component of propolis that has a variety of potential pharmacological effects. Although we previously demonstrated that propolis has antidepressant-like activity, the effect of CAPE on this activity remains unknown. The present study assessed whether treatment with CAPE (5, 10, and 20 *µ*mol/kg for 21 days) has an antidepressant-like effect in mice subjected to chronic unpredictable stress via tail suspension (TST) and forced swim (FST) tests. CAPE administration induced behaviors consistent with an antidepressant effect, evidenced by decreased immobility in the TST and FST independent of any effect on serum corticosterone secretion. Western blots, conducted subsequent to behavioral assessment, revealed that CAPE significantly decreased glucocorticoid receptor phosphorylation at S234 (pGR(S234)), resulting in an increased pGR(S220/S234) ratio. We also observed negative correlations between pGR(S220)/(S234) and p38 mitogen-activated protein kinase (p38MAPK) phosphorylation, which was decreased by CAPE treatment. These findings suggest that CAPE treatment exerts an antidepressant-like effect via downregulation of p38MAPK phosphorylation, thereby contributing to enhanced GR function.

## 1. Introduction

Depression is one of the most common and serious mental health problems in society today. The pathophysiology of depression is postulated to involve functional alternations in glucocorticoid receptor (GR) signaling. Clinical studies have shown that cortisol excess (hypercortisolism) is often found in patients with depression, which has attributed to impaired feedback regulation of the hypothalamus-pituitary-adrenal (HPA) axis [1, 2]. This is possibly caused by impaired GR function in the hippocampus, hypothalamus, and pituitary gland [3]. In line with this, human postmortem studies have shown that GR expression (mRNA and protein) in the hippocampus is downregulated in suicide victims with a history of childhood abuse [2, 4, 5]. In addition to decreased GR expression, a recent study reported that a change in the phosphorylation status of GRs is involved in the pathogenesis of depression. Studies of leukocyte GRs in patients with episodes of major depressive disorder (MDD) have demonstrated that increased phosphorylation of GR at S226 and, to a lesser extent, at S211 result in a decreased pGR(S211)/(S226) ratio [6]. Therefore, the pathophysiology of depression may be also influenced by alternations in the phosphorylation status of GRs.

In our previous investigations, an ethanol extract of honeybee propolis had a strong antidepressant-like effect in mice subjected to the forced swim test (FST), in which the extract (i) modulated differential phosphorylation of GRs, between S220 and S234, thereby increasing the pGR(S220)/(S234) ratio, and (ii) restored phosphorylation of the cAMP-responsive element binding (pCREB) protein at S133 [7]. These results imply that GR phosphorylation is involved in the antidepressant-like activity of propolis. Propolis contains more than 200 natural constituents, including polyphenols, phenolic aldehydes, sesquiterpene-quinones, coumarins, amino acids, fatty acids, steroids, and inorganic compounds [8]. Among them, caffeic acid phenethyl ester (CAPE) as a representative component of propolis has shown pharmacological effects on the central nervous system [9]. For example, CAPE has been shown to have a protective effect after focal cerebral ischemia in rats [10, 11] and rabbits [12]. CAPE is also reported to protect cultured cerebellar granule neurons against glutamate-induced neurotoxicity [13]. Furthermore, a recent study has reported that CAPE directly activates CREB [14], as seen in our previous study with propolis, exhibiting a neuroprotective effect* via* brain-derived neurotrophic factor (BDNF), a target gene of CREB signaling [15]. From these observations, CAPE would be expected to have ameliorative activity toward depression via modulating GR signaling.

In the present study, CAPE was investigated for its antidepressant-like effects in mice subjected to chronic unpredictable stress (CUS). Biochemical analysis was also performed to investigate GR function by detecting the pGR(S220)/(S234) ratio in three subfamilies of mitogen-activated protein kinases (MAPKs), including extracellular signal-regulated kinases (ERKs), c-Jun amino-terminal kinases (JNKs), and p38 MAPKs, which represent the main signaling pathway regulating GR function [16, 17].

## 2. Materials and Methods

### 2.1. Animals

Six-week-old CD-1 male mice (Orient Co., Seoul, Korea) were housed (five per cage) for 1 week before the start of experiments in a temperature-controlled (22–24°C) room with a 12 h light/dark cycle. The lights were on between 08:00 h and 20:00 h. Mice were given at least 1 week to acclimatize to their environment prior to the onset of the experiments. The Institutional Animal Care and Use Committee of the Korea Basic Science Institute (KBSI) reviewed and approved the present study (KBSI-AEC 1109). All animal procedures were conducted in accordance with the “Guide for the Care and Use of Laboratory Animals” issued by the Laboratory Animal Resources Commission of KBSI.

### 2.2. Stress Procedure and Drug Administration

Mice were subjected to CUS twice a day (AM and PM) for 21 consecutive days as described by Ducottet's group with a slight modification [18]. The following stressors were used: 2 h restraint, 24 h illumination, 24 h food deprivation, 12 h water deprivation, 2 min cold swimming (at 4°C), 2 min hot swimming (at 45°C), 24 h wet sawdust, 0.5 mA foot shock (maximum shock duration of 10 s), inversion of the light/dark cycle, 1 min tail pinch (1 cm from the tip of the tail), and 12 h cage tilting. For each day of CUS, mice were randomly assigned AM and PM stressors.

Based on our preliminary study and previous study [19] in which treatment with only CAPE showed no significant change in depressive-like behavior, mice were randomly divided into six groups (*n* = 9 per group): naive group, nonstressed model with vehicle treatment; vehicle (VEH) group, CUS model treated with vehicle (1% dimethyl sulfoxide/corn oil); three CAPE groups, CUS model treated with CAPE (5, 10, and 20 *μ*mol/kg in vehicle solution; Sigma, St. Louis, MO, USA) [20–22]; and fluoxetine (FLX) group, CUS model treated with FLX (10 mg/kg, dissolved in saline; provided by Daewoo Pharmaceuticals, Pusan, Korea). All drugs and vehicle were administered intraperitoneally (i.p.) in a volume of 1 mL/kg body weight at 09:00 h each day.

### 2.3. Forced Swim Test

The modified FST described by Petit-Demouliere and colleagues [23] was used in this study. Briefly, mice were forced to swim for 6 min in a glass beaker (diameter, 13 cm; height, 19 cm) filled to a depth of 14 cm with 23–25°C water. A mouse was considered immobile when it floated without attempting to swim. During the 6 min test session, the last 4 min spent immobile was measured by two trained observers who were blind to the treatment.

### 2.4. Tail Suspension Test

The tail suspension test (TST) was carried out according to a previously described method [24]. In brief, each mouse was suspended on the edge of a lever 58 cm above a tabletop using adhesive tape placed approximately 1 cm from the tip of the tail. A mouse was considered immobile when it hung passively and was completely motionless. The total duration of immobility was measured over a 6 min period by blinded observers.

### 2.5. Corticosterone Assay

The corticosterone assay has been described previously [25]. Corticosterone serum levels (*n* = 7–9 per group) were measured using a commercially available enzyme immunoassay (EIA) kit (R&D Systems, Inc., Minneapolis, MN, USA), according to the manufacturer's instructions.

### 2.6. Western Blot Analysis

The Western blot analysis of extracts of frozen hippocampal tissue (*n* = 9 per group) was performed as described previously [26]. Protein concentrations were determined using a bicinchoninic acid (BCA) protein assay (Pierce Biotechnology, Rockford, IL, USA), and the primary antibodies used were specific for GR (1 : 1,000; Santa Cruz Biotechnology, Santa Cruz, CA, USA), phospho-GR (S220) (1 : 500; Cell Signaling Technology, Inc., Beverly, MA, USA), phospho-GR (S234) (1 : 500; Abcam, Cambridge, UK), p38 MAPK (1 : 500; Cell Signaling Technology), phospho-p38 MAPK (Tyr182) (1 : 500; Santa Cruz Biotechnology), and *β*-actin (1 : 10,000; Sigma). Detection was achieved using horseradish peroxidase-conjugated IgG (1 : 5,000; Santa Cruz Biotechnology) and visualized using an electrochemiluminescence (ECL) assay kit (Amersham, Little Chalfont, Buckinghamshire, UK). Band intensities were determined using the ImageJ program (open source ImageJ software available at http://rsb.info.nih.gov/ij/).

### 2.7. Statistical Analysis

Data were analyzed using a one-way analysis of variance (ANOVA), followed by the Sadik post hoc test, using Prism 4 (GraphPad Software, Inc., San Diego, CA, USA) for multigroup comparisons. Pearson's correlation coefficients were calculated for each pGR(S220)/(S234) ratio, immobility time, and p-p38MAPK/p38MAPK ratio. The level of statistical significance was set at *P* < 0.05. Results are expressed as means ± standard errors of the mean (SEM).

## 3. Results

### 3.1. Antidepressant-Like Behavioral Effect of CAPE

**Figure 1:**
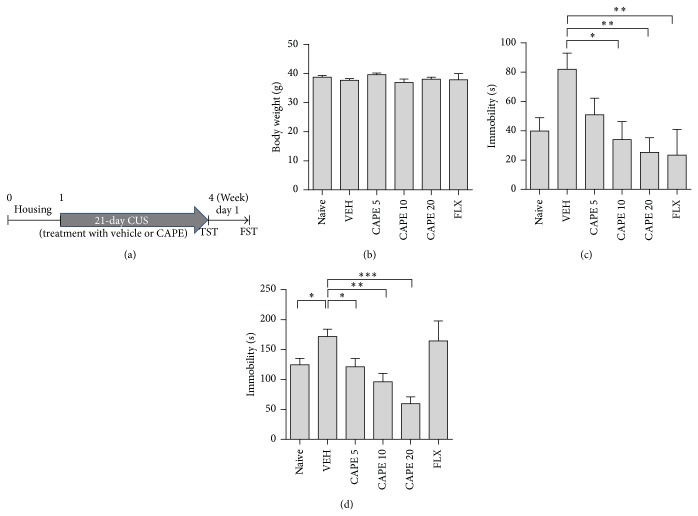
Effects of CAPE on depression-like behaviors during the TST and FST. (a) Experimental schematic of the antidepressant-like activity of CAPE. Mice were subjected to chronic unpredictable stress (CUS) for 21 consecutive days following intraperitoneal injection with vehicle (VEH), CAPE (5, 10, and 20 *μ*mol/kg), or fluoxetine (FLX, 10 mg/kg). Body weight was measured prior to the behavioral test (b). Time spent immobile was recorded during the TST (c) and FST (d). The columns and error bars represent means ± SEM (*n* = 9 per group). Data were analyzed using a one-way ANOVA, followed by Tukey's post hoc test. ^*^ *P* < 0.05, ^**^ *P* < 0.01, and ^***^ *P* < 0.001 versus VEH.

At the beginning of the experiment, the antidepressant-like activity of CAPE was assessed in mice subjected to CUS by measuring immobility time during the FST and TST (Figure 1(a)). A significant effect of CAPE treatment was observed in the TST (*F*_5,48_ = 4.59, *P* < 0.01) and the post hoc test showed that CAPE treatment significantly decreased immobility time in a dose-dependent manner (Figure 1(c)). In the FST (*F*_5,48_ = 11.77, *P* < 0.01), mean immobility time of the CAPE treatment group also decreased in a dose-dependent manner. Fluoxetine (10 mg/kg), used as the positive control for antidepressant-like activity, markedly decreased immobility time in the TST but not in the FST.

### 3.2. Effect of CAPE on Serum Corticosterone Levels

**Figure 2:**
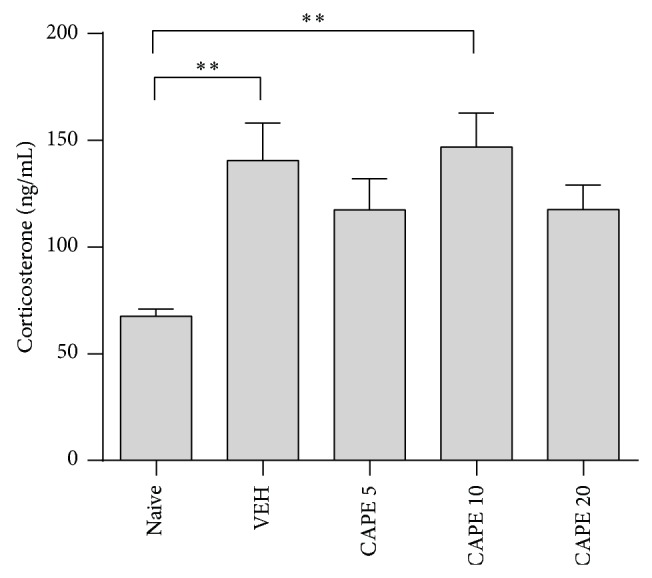
Effect of CAPE treatment on serum corticosterone levels in mice exposed to CUS. Trunk blood was collected 30 min after the FST. The columns and error bars represent means ± SEM (*n* = 9 per group). Data were analyzed using a one-way ANOVA, followed by Tukey's post hoc test. ^*^ *P* < 0.05 versus VEH.

Although a significant difference in the basal level of corticosterone was observed among groups (Figure 2; *F*_4.40_ = 4.56, *P* < 0.01), a difference was not found between CAPE-treated and VEH groups, suggesting that CAPE treatment did not influence corticosterone secretions under chronic stress. Basal levels of corticosterone in the CUS group were significantly increased compared to the nonstressed naive group.

### 3.3. Effect of CAPE on GR Phosphorylation

**Figure 3:**
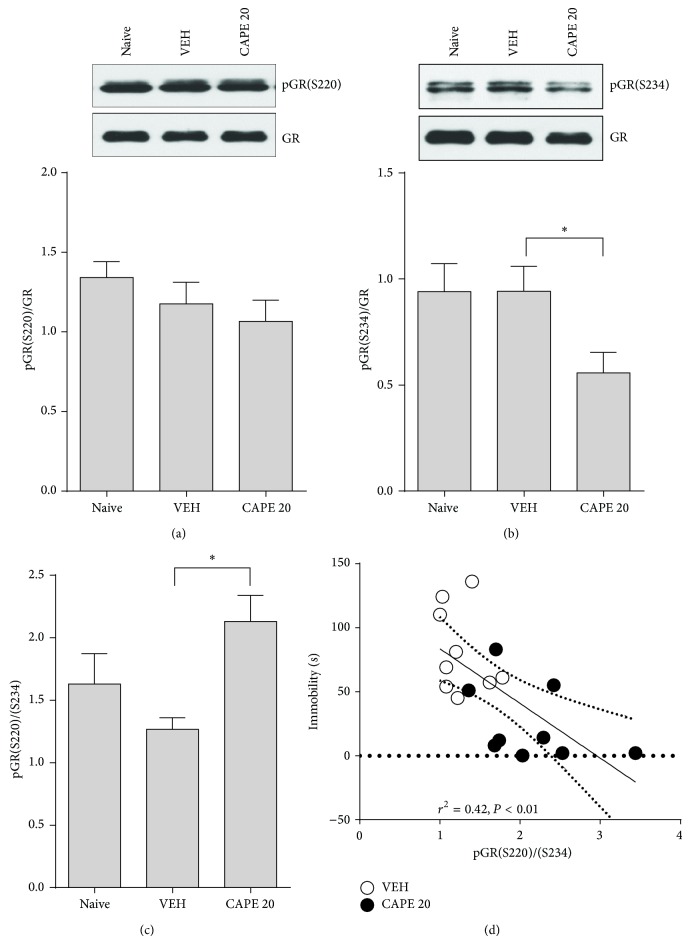
Differential GR phosphorylation by CAPE treatment. Following the FST, the hippocampus was dissected and frozen in dry ice. (a) Representative Western blot images (upper panel) and (b) quantitation (lower panel) depicting pGR(S220), pGR(S234), and GR expression in hippocampal lysates. The average band intensity of the pGRs was normalized by total GR protein. (c) The pGR S220/S234 ratio. The columns and error bars represent means ± SEM (*n* = 9 per group). Data were analyzed using a one-way ANOVA followed by Tukey's post hoc test. ^*^ *P* < 0.05 versus VEH. (d) Pearson correlation analysis between the pGR(S220)/(S234) ratio and time spent immobile in the TST. Scatter plots with fitted linear regression lines depict significant correlations. Each of the eight points corresponds to VEH (open circle) and CAPE 20 *μ*mol/kg (closed circle), respectively. The squared correlation coefficient (*r* ^2^) is provided above the plot.

Our previous study demonstrated that enhanced GR function, via differential phosphorylation between S220 and S234, was associated with antidepressant-like activity of the propolis extract [7]. Therefore, we examined phosphorylation status at S220 and S234 to investigate whether CAPE treatment could influence GR function [27]. Western blot analysis with whole hippocampal lysates showed no significant effect on pGR(S220) in the CAPE-treated group (Figure 3(a); *F*_2,24_ = 1.24, *P* = 0.41). In contrast, pGR(S234) was significantly lower in the CAPE-treated group compared to the VEH group (Figure 3(b); *F*_2,24_ = 3.64, *P* < 0.05). As a result, the ratio of pGR(S220)/(S234) was significantly different between groups (*F*_2,24_ = 5.07, *P* < 0.05), where the CAPE-treated group displayed a 220% increase in the ratio compared to the VEH group (Figure 3(c)). Moreover, a significant negative correlation was found between the ratio and immobility time in the TST (Figure 3(d), *r* ^2^ = 0.42, *P* < 0.01). This indicates that a higher pGR(S220)/(S234) ratio was associated with antidepressant-like behavioral effects of CAPE treatment.

### 3.4. Reduced p38 MAPK Phosphorylation and the Relationship between p-p38 MAPK and the pGR(S220)/(S234) Ratio

**Figure 4:**
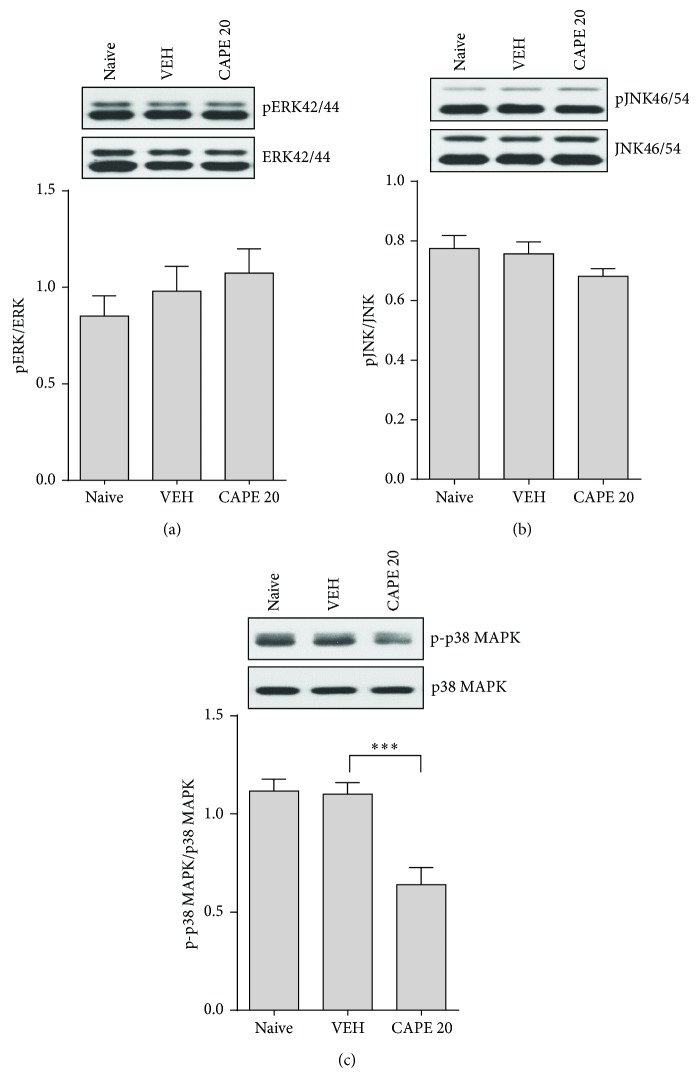
Differential effect of CAPE on MAPK phosphorylation in the hippocampus. Representative Western blot images (upper panel) depicting the expression of phosphorylation of (a) ERK, (b) JNK, and (c) p38MAPK in hippocampal lysates. Average band intensities (lower panel) were normalized to the corresponding nonphosphorylated MAPKs. The columns and error bars represent means ± SEM (*n* = 9 per group). Data were analyzed using a one-way ANOVA followed by Tukey's post hoc test. ^***^ *P* < 0.001 versus VEH.

**Figure 5:**
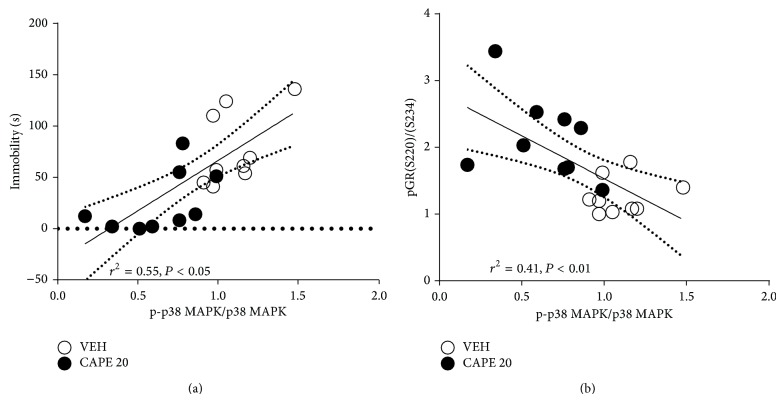
Pearson correlation analysis of p38MAPK phosphorylation and time spent immobile in the (a) TST and (b) pGR(S220)/(S234) ratio. Each of the eight points corresponds to VEH (open circle) and CAPE 20 *μ*mol/kg (closed circle). Lines represent the linear regression fit. The squared correlation coefficient (*r* ^2^) is provided above the plot.

MAPKs such as ERK, JNK, and p38MAPK phosphorylate GR and thereby regulate its transcriptional activity. Therefore, we next examined the activation of these three MAPKs to investigate possible associations with the antidepressant-like activity of CAPE. Western blot analysis revealed that CAPE treatment had no effect on the activation of ERK1/2 and JNK in the hippocampus of mice subjected to CUS (Figures 4(a) and 4(b); *F*_2,24_ = 0.85, *P* = 0.44 for ERK1/2; *F*_2,24_ = 1.82, *P* = 0.19 for JNK1/2). However, a significant inhibiting effect was found for p-p38MAPK (Figure 4(c); *F*_2,24_ = 15.01, *P* < 0.01). ERK1/2, JNK, and p38MAPK expression was not significantly different between groups (data not shown), and a correlation analysis revealed that p38MAPK activation was associated with depressive behavior (Figure 5(a); *r* ^2^ = 0.55, *P* < 0.05). Furthermore, a strong negative linear correlation was found between p38MAPK activation and the pGR(S220)/(S234) ratio (Figure 5(b); *r* ^2^ = 0.41, *P* < 0.01). These data suggest that enhanced GR function is involved in the antidepressant-like activity of CAPE via a downregulation of p38 signaling.

## 4. Discussion

We examined the antidepressant-like effect of CAPE, which is the main component of propolis. We previously demonstrated antidepressant-like properties of crude propolis extract via enhanced GR function in the hippocampus, as reflected by an increase in the pGR(S220)/(S234) ratio [7]. Consistent with those results, CAPE in the present study was found to attenuate depressive behavior in the TST and FST and also increased the pGR(S220)/(S234) ratio. Moreover, we found a role for p38MAPK signaling in the modulation of GR function. Specifically, we revealed that phosphorylation of p38MAPK was negatively correlated with the pGR(S220)/(S234) ratio and positively correlated with depressive behavior. These findings suggest that CAPE restores GR function via p38MAPK, which may be associated with improvements in depressive behavior.

We used fluoxetine as a positive control in CD-1 mice exposed to CUS in order to confirm that our animal model was suitable for the investigation of a possible antidepressant effect of CAPE. Although CAPE treatment significantly attenuated CUS-induced depression-like behavior in both the FST and TST, fluoxetine treatment yielded inconsistent results across the two tests. Both the FST and TST are widely used as behavioral screens in rodents and are sensitive to and selective for clinically effective antidepressant drugs. However, differential sensitivity to the immobility-reducing effects of various antidepressants, including characteristics of dose-response curves, has previously been reported [28]. For example, in one study, the antidepressant imipramine exhibited a U-shaped dose-response function in the FST but a linear pattern of activity in the TST over the same dose range [29]. Therefore, our inconsistent results for the FST and TST may be explained by the different characteristics of the dose-response curves. The optimal dose of a positive control drug should be determined prior to the screening of novel antidepressant drugs, particularly with respect to the FST. Another explanation as to why we saw no response to fluoxetine treatment could be due to a difference in mouse strains. Differences may, in part, be derived from the diverse genetic backgrounds of the various strains, and this would be critical in determining baseline performance and sensitivities to different types of antidepressant drugs. Consistent with our fluoxetine results, Lucki's group reported no effect of fluoxetine on behavioral immobility in CD-1 mice [30]. However, CD-1 mice can be useful when investigating whether a treatment has antidepressant-like activity [23].

It is also interesting to note that no effect of CAPE treatment was seen on corticosterone secretion in this study, whereas in the previous study propolis significantly inhibited corticosterone secretion [7]. One possibility for the different regulation of corticoid secretion may be due to the discrepancy between acute and chronic stress models. More specifically, in our previous study we used an acute stress model, while we employed a chronic stress model in the current study. In line with this, it is well known that the HPA axis is activated in response to acute stress, which is suppressed by antidepressant treatment [31]. In contrast, no significant variations in hormone levels occur after chronic treatment of antidepressants in mild chronic stress [32, 33]. Nevertheless, we cannot exclude that other components of propolis (aside from CAPE) affect the secretion of corticosterone.

To determine the molecular basis of the antidepressant-like effects of CAPE, we investigated changes in corticosterone secretion, GR expression, and phosphorylation status, which are the main neurobiological factors that influence GR function. It is widely believed that GR dysfunction is involved in the pathogenesis of depression [34]. Furthermore, antidepressant treatment has been reported to normalize HPA axis activity by increasing GR expression and function [35], suggesting that improved GR signaling may represent an important target for antidepressants [36]. In accordance with this notion, we previously demonstrated that the antidepressant-like properties of propolis [7] and Cortex Mori Radicis extract [25] are associated with enhanced GR function via an increase in the pGR(S232)/(S246) ratio. In the present study, we also observed enhanced GR function via CAPE treatment, as evidenced by the increased pGR(S232)/(S246) ratio. Furthermore, the ratio was negatively correlated with time spent immobile in the TST, suggesting that this ratio might be a neurobiological biomarker of response to antidepressant treatment. Interestingly, however, levels of corticosterone were not affected by CAPE treatment in the present study, whereas our previous study using propolis demonstrated suppression of corticosterone secretions. These results can be explained by a mechanism of ligand-independent GR activation, induced by *β*_2_-adrenergic receptor agonists, which have anti-inflammatory actions [37]. In support of this concept, some antidepressants have proven effective even without restoring altered HPA axis responsivity [38]. Therefore, it may be instructive to investigate whether CAPE induces GR activation in the absence of hormones* in vitro*.

Many preclinical studies have demonstrated that chronic antidepressant treatment activates the MAPK signaling cascade [39] in which p38MAPK modulates GR function via the three major sites on its N terminus [17]. The results of our current study also showed that p38MAPK signaling is associated with the antidepressant-like activity of CAPE, where CAPE treatment decreased p38MAPK phosphorylation. Moreover, p38MAPK phosphorylation was negatively correlated with the pGR(S220)/(S234) ratio, suggesting that inhibition of p38MAPK phosphorylation in the hippocampus may play a key role in GR activity. Nevertheless, we cannot exclude the involvement of other signaling pathways in the pharmacological action of antidepressants, since a large body of data has accumulated suggesting that antidepressants activate various pathways including MAPK [40, 41], serine/threonine-specific protein kinase (AKT, [42]), calcium/calmodulin-dependent kinase (CaMK, [43, 44]), and cAMP response element binding protein (CREB, [45–47]) pathways. Particularly, a decrease in mRNA and protein expression of ERK1/2 via decreased p44/42 MAP kinase activity has been reported in the hippocampus of victims of suicide with major depression [48]. Functional changes in ERK1/2 signaling by chronic treatment with fluoxetine were also reported in rats [49], indicating that a dynamic modulation of ERK1/2 is involved in the pathophysiology and therapeutic effects of depression. It is also interesting to note that cross-talk between site-specific phosphorylation and MAPK cascades plays an important role in GR function [25, 50–52] and that GR is coupled to the activation of CaMKII-BDNF-CREB pathways to mediate memory consolidation [53]. Further, dysfunction of GR function leads to alterations in the sensitivity of CREB to antidepressant actions via CaMK signaling [54], suggesting a possible role of GR signaling in the therapeutic effects of depression. Thus, it is tempting to speculate how CAPE treatment might influence CaMK signaling via GR function.

In conclusion, the present study is the first to demonstrate the antidepressant-like activity of CAPE, which may be mediated by enhanced GR function that is, in-turn, linked to p38MAPK signaling. Although further investigation is required to identify ligand-independent mechanisms of GR function, our findings suggest that CAPE might represent a novel pharmacological option for patients with neuropsychiatric disorders, including major depression.
